# Coal and Gangue Separating Robot System Based on Computer Vision

**DOI:** 10.3390/s21041349

**Published:** 2021-02-14

**Authors:** Zhiyuan Sun, Linlin Huang, Ruiqing Jia

**Affiliations:** 1 School of Mechanical Electronic & Information Engineering, China University of Mining & Technology, Beijing 100083, China; zqt1800402042g@student.cumtb.edu.cn; 2 Mine Robot Research Center, China University of Mining & Technology, Beijing 100083, China; 108207@cumtb.edu.cn

**Keywords:** coal and gangue, YOLO, detection, robot

## Abstract

In coal production, the raw coal contains a large amount of gangue, which affects the quality of coal and pollutes the environment. Separating coal and gangue can improve coal quality, save energy, and reduce consumption and make rational use of resources. The separated gangue can also be reused. Robots with computer vision technology have become current research hotspots due to simple equipment, are efficient, and create no pollution to the environment. However, the difficulty in identifying coal and gangue is that the difference between coal and gangue is small, and the background and prospects are similar. In addition, due to the irregular shape of gangue, real-time grasping requirements make robot control difficult. This paper presents a coal and gangue separating robot system based on computer vision, proposes a convolutional neural network to extract the classification and location information, and designs a robot multi-objective motion planning algorithm. Through simulation and experimental verification, the accuracy of coal gangue identification reaches 98% under the condition of ensuring real-time performance. The average separating rate reaches 75% on low-, medium-, and high-speed moving conveyor belts, which meets the needs of actual projects. This method has important guiding significance in detection and separation of objects in complex scenes.

## 1. Introduction

In coal production, the raw coal mined directly from coal mines contains a lot of gangue, which contains a lot of heavy metals, and affects the calorific value of coal, causing serious pollution to the environment [1]. Therefore, it is essential to separate coal and gangue to make them play their respective roles [2,3]. As an important part of the coal industry, coal gangue separation can improve coal quality, save energy, and reduce consumption and thus rationally use resources [4]. At present, coal and gangue separation includes moving sieve jigging method, heavy medium separation, dual-energy γ-ray separation, X-ray, and image recognition method. The working principle of moving sieve jigging method in gangue sorting is a follows: sieve mechanism moves up and down in an aqueous medium by some kind of power-driven machine, raw coal moves with the media water, and then, coal and gangue forms stratified layers according to their different densities [5]. The principle of heavy-medium gangue separation is to use a liquid with a density between coal and gangue as the medium. If the density of coal is lower than the medium, it will float up, and the gangue will sink if the density is higher than that of the medium to achieve the purpose of separation [6]. However, moving sieve jigging method and heavy medium separation are mechanical methods and therefore, can only deal with gangue within a specific size range with huge equipment, high cost, and high energy consumption. Mechanical methods also cause environmental pollution in the process. The dual-energy γ-ray gangue sorting is composed of a low energy source and a middle energy source, which form a radiation excitation field. When two rays pass through the raw coal at the same time, different attenuation amplitudes are generated, because the coal and gangue have different radiation absorption capabilities. The density is calculated by the attenuation amplitude to judge the classification [7]. Radioactive separating uses radioactive sources, which have strict requirements for radiation protection [8]. In addition, manual separation must cooperate with some above methods in the follow-up. Manual separating is widely used because it can be applied to every link of the sorting process, not only to identify but also to separate coal and gangue; although workers continue to perform mechanical repetitive work on the production line, the sorting efficiency does not even reach 50%, because there are many missed and wrong sorts.

In order to liberate workers from such dirty, dangerous, and dull (3D) work, a wave of research on industrial robots has been set off. The changes in external information are perceived through vision and fed back to the robot controller, so its application fields will be more extensive [9,10]. Shang proposes a Delta parallel coal gangue online sorting robot based on image recognition and an improved comprehensive calibration method, which avoids the influence of robot installation error on grasping accuracy [11]. Li proposes an image processing-based method and further develops a positioning and identification system [12]. It is worth mentioning that there is no robot for dynamic sorting of multi-target scenarios in previous work [13]. Vision technology can expand the scope of application of robotics, and the interaction between the two can improve the level of industrial production and promote social change [14]. In terms of coal and gangue recognition, people began to use traditional and deep learning methods to extract the characteristics of coal and gangue [15,16,17]. Traditional methods of manually extracting the gray, texture, and edge contour of coal and gangue have achieved certain results. Lepisto introduced a rock texture classification method, based on the textural and spectral features of the rock. Reddy separated gangue from coal based on histogram thresholding [18]. Zhang used energy, contrast, correlation, and entropy as the feature vector to realize the automatic identification of coal and gangue according to the features with large differences in texture feature values of coal and rock, and the entropy value had the best identification effect [19]. The above coal and gangue separating methods mainly rely on manual extraction of feature values with dependence on experience and knowledge, which are subjective and limited [20]. With the continuous development of deep learning in image recognition, more and more algorithms have begun to be applied to coal and gangue recognition [21,22]. The deep learning algorithm can avoid manual extraction of features and the deep learning algorithm relies on a large amount of data for training [23]. The deeper the convolution level, the more buried information can be found. Alfarzaeai built a new model called (CGR-CNN) based on convolutional neural network (CNN) using thermal images as standard images for coal and gangue recognition [24]. Pu employed a convolutional neural network (CNN) to recognize coal and gangue images and help segregate coal and gangue [25]. However, the studies only identified the type of picture, and did not give the location of the object, so subsequent sorting was not possible. Target detection can be seen as a combination of image classification and positioning. This scene is more suitable for target detection of coal and gangue. Wang automatically extracts image features of coal and gangue to obtain feature maps, and then extracts candidate regions of coal or gangue through RPN (Region Proposal Network) [26]. Li designed CG-RPN to generate the target area of candidate objects, which can detect multiple coals and gangue in an irregular arrangement for simple background, and a relatively large distance between the target [9]. Many studies in the laboratory have achieved good accuracy in image recognition for only a single object (gangue or coal) in the image. In fact, the number and class of targets in the picture are uncertain, which makes the precise location of coal and gangue more complicated than general classification tasks. Meanwhile, the object (coal and gangue) is highly similar to the background (black belt), which greatly increases the difficulty of detection, and the detection of dynamic targets will also affect the accuracy. Even if the target is detected, due to the irregular shape of the objects, it is very difficult to extract and capture features [27,28]. The strategy of sorting as many objects as possible in the shortest possible time requires reasonable multi-target motion planning.

In this article, we propose a dynamic target detection method called CG-YOLO to identify the classification and location of coal and gangue. In this method, the target boxes in the images are clustered to form anchor boxes with different scales through the K-Means algorithm. Additionally, the improved Darknet network suitable for coal and gangue detection is designed for training images. It is confirmed that CG-YOLO have both real-time and accuracy in detection. Then, we design a six-axis robotic arm system, which is composed of the six-axis robotic arm body, control system, host computer, and stabilized power supply. In order to meet the separating strategy, motion planning is carried out according to the result of target detection, including the position, size, and confidence of coal and gangue. After completing the trajectory planning, the grasping task is assigned to the corresponding robotic arm controller. After the robotic arm acquires the task, it dynamically monitors the target according to the acquired task. When the target enters the robotic arm workspace, the robotic arm is driven to complete the gangue grasping. We build models of image acquisition devices, objects to be identified, conveyor belts, and robotic arms in simulation software CoppeliaSim, and through remote API, the system completes the identification and sorting process in the simulation environment. Then in real experiment, the robot is tested in high, medium, and low speeds targets. The main contributions of the work in this paper are the following: (1) propose a dynamic target detection method and establish a data set of coal and gangue, (2) propose a movement planning method in line with the coal gangue sorting strategy, and (3) control the robot to complete the sorting task.

The paper is structured as follows: Section 2 proposes the method of coal and gangue detection and motion planning of robot. Simulation and experiment and results are monitored and discussed in Section 3. Section 4 discusses the possibility and prospects of the application of artificial intelligence technology in the coal field, and Section 5 concludes this work.

## 2. The Proposed Method

In this section, we present the construction method of the data set, network and training of the target detection model, and the robot’s motion planning strategy for multi-target tasks.

### 2.1. Sample and Data

The number and richness of samples have a significant impact on training results [26]. The images were taken by Delsa’s G3-GM10-M2590 industrial Camera and VS1614-10 m lens. The samples included coal, gangue, and mix (both coal and gangue). Considering the objects at different speeds, images with the conveyor speed at 0–100 mm/s were collected. Images were collected at three typical speeds: low (10 mm/s), medium (50 mm/s), and high (100 mm/s). The sample set contained 2020 pictures. The distribution is shown in Table 1. It is worth mentioning that each picture contained multiple objects.

The collected images were manually annotated. There were two predefined classes, coal and gangue, and objects less than three-fourth were ignored when labeling. Each image corresponded to its annotation file, which contained the location and class information. Among the dataset, there were 5034 gangue objects and 3125 coal objects in total.

There were two types of objects in the established data set, which had big differences within the class. The objects in the input images were clustered by K-Means clustering algorithm; then, six anchors were acquired, which were (23, 41), (53, 30), (42, 82), (90, 83), (102, 115), and (164, 158). These anchor frames could achieve a larger IOU with the target on larger scale and smaller scale. For a target that was too small, i.e., cinder, it was ignored in the annotate process, so it was also not considered in the anchor frame clustering process.

### 2.2. Model Construction and Training

#### 2.2.1. Network

**Figure 1:**
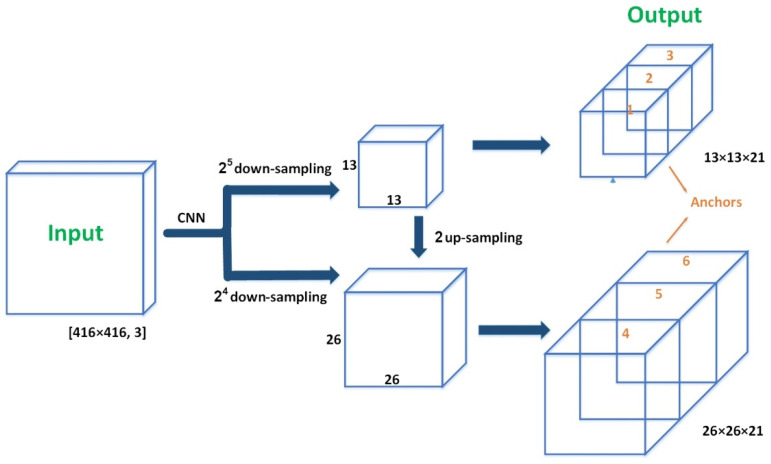
The network structure of CG-YOLO.

CG-YOLO is based on the deep learning framework Darknet [29,30,31], which is used to extract features. The backbone network contains a total of 75 layers, of which 53 are convolutional layers and the rest are res layers. The characteristic interaction layer of YOLO network is 75–105 layers. In order to detect objects of size, the input image was down-sampled five times to get different scale. The result of the fifth down-sampling was outputted, and then, this result was up-sampled to integrate the result of the fourth down-sampling as the output of another scale. When the input image was 416 × 416, the outputs were 13 × 13 and 26 × 26 through CNN. The network structure of CG-YOLO is shown in Figure 1.

The detection object is found by the anchor boxes, and then, the position relative to the anchor box (*t_x_*, *t_y_*, *b_h_*, and *b_w_*) of each prediction box, where center coordinates *t_x_* and *t_y_*, box height *b_h_* and width *b_w_*, a target confidence level *P_obj_*, and the class of object of the anchor are returned. The information *C* contained in each cell is as follows:

(1) C=3×(5+N),

in which *N* is the number of classes. Each cell of CG-YOLO returns *C* = 21.

**Figure 2:**
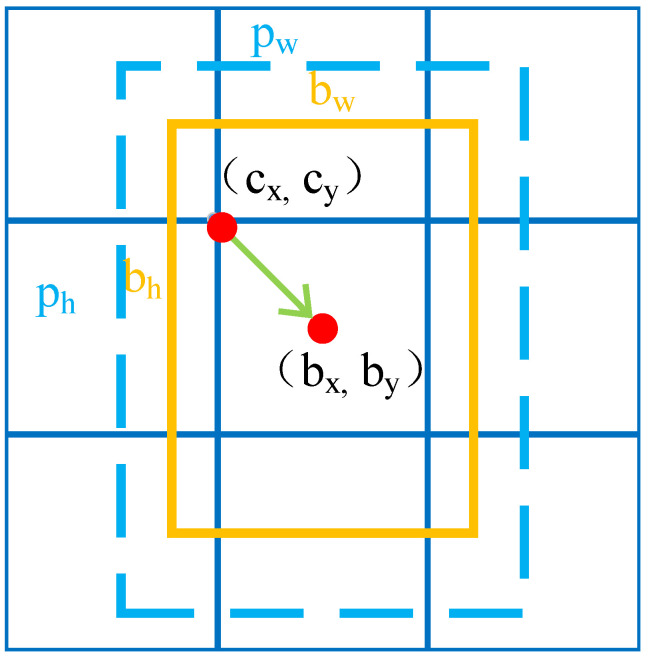
Position offset and width and height stretch.

However, the anchor box does not match the object in the image, so it needs to be adjusted to become a prediction box, the process is shown in Figure 2. First, the initial position of the anchor box (*c_x_*, *c_y_*) is the position of the cell in 13 × 13 or 26 × 26, which is adjusted by offset according to the returned (*t_x_*, *t_y_*). Then, the width and height of the anchor (*p_w_*, *p_h_*) are adjusted according to the returned (*t_w_*, *t_h_*). Finally, a prediction box (*b_x_*, *b_y_*_,_ *b_w_*, and *b_h_*) is formed, and the formula is as follows:

(2) $$\begin{array}{l} b_{x}=c_{x}+\sigma \left(t_{x}\right)\\ b_{y}=c_{y}+\sigma \left(t_{y}\right)\\ b_{w}=p_{w}e^{t_{w}}\\ b_{h}=p_{h}e^{t_{h}} \end{array}$$

in Equation (2): $\sigma \left(x\right)=\frac{e^{x}}{1+e^{x}}$.

#### 2.2.2. Training Process

**Figure 3:**
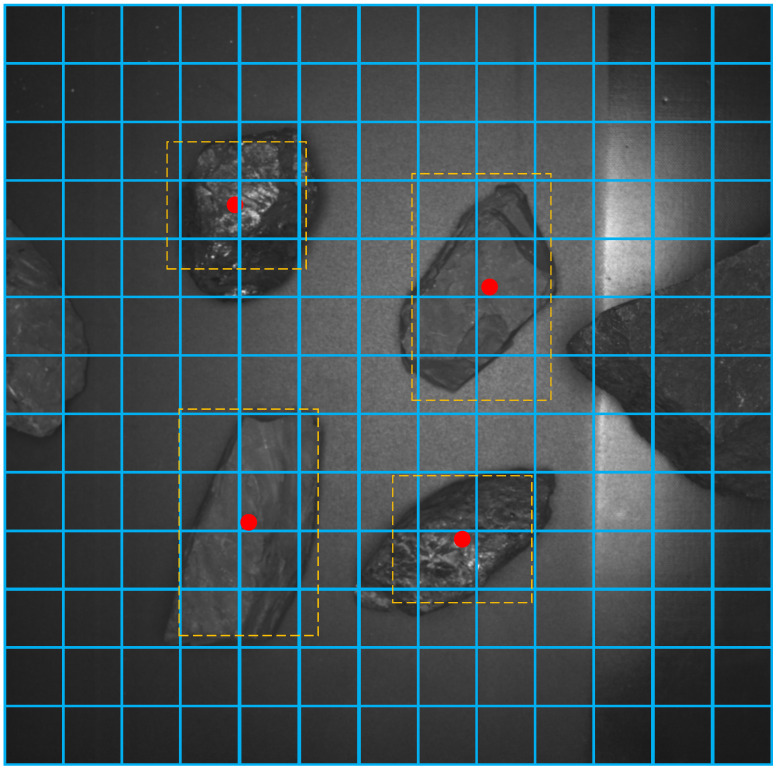
Matching and annotation of real frame.

The learning goals of training are the offsets of *t_x_*, *t_y_*, *b_h_*, and *b_w_*, adjusting the prediction through the supervision information of the ground truth box. The ground truth box (*gt_x_*, *gt_y_*, *gt_w_*, and *gt_h_*) is artificially labeled, in which (*gt_x_*, *gt_y_*) is the actual center position and (*gt_w_* and *gt_h_*) is the width and height of the box, respectively. The cell where the center of object is located is responsible for detecting this object, and the anchor with the largest IoU matches this object, as shown in Figure 3. The calculation of supervision information (t_x_*, t_y_*, t_w_*, and t_h_*) is shown in the following formula:

(3) $$\begin{array}{l} \sigma \left(t_{x}{}^{*}\right)=gt_{x}-c_{x}\\ \sigma \left(t_{y}{}^{*}\right)=gt_{y}-c_{y}\\ t_{w}{}^{*}=log(\frac{gt_{w}}{p_{w}})\\ t_{h}{}^{*}=log(\frac{gt_{h}}{p_{h}}) \end{array}$$

For the calculation of the loss, overall loss function is calculated into three parts. First classify the prediction frame, with the real frame falls on, without the real frame falls on, and the frame that does not match but with relatively large IoU. The first one is a positive example, we calculate the location loss, objectness loss, and classification loss. The second is a negative example, return the objectness of the prediction box expected to 0. The third is a non-positive and non-negative example, and its loss function is not calculated. The overall loss function is as follows:

(4) $$\begin{array}{ll} L\mathrm{oss} & =\sum_{i=0}^{S^{2}}\sum_{j=0}^{B}\exists_{i,j}^{obj}[{\left(x_{i}-\hat{x_{i}}\right)}^{2}+{\left(y_{i}-\hat{y_{i}}\right)}^{2}]+\sum_{i=0}^{S^{2}}\sum_{j=0}^{B}\exists_{i,j}^{obj}[{\left(w_{i}-\hat{w_{i}}\right)}^{2}+{\left(h_{i}-\hat{h_{i}}\right)}^{2}]\\ & +\sum_{i=0}^{S^{2}}\sum_{j=0}^{B}\exists_{i,j}^{obj}{\left(C_{i}-\hat{C_{i}}\right)}^{2}+\sum_{i=0}^{S^{2}}\sum_{j=0}^{B}\exists_{i,j}^{nobj}[{\left(C_{i}-\hat{C_{i}}\right)}^{2}\\ & +\sum_{i=0}^{S^{2}}\exists_{i}^{obj}\sum_{c\in classes}{\left(p_{i}(c)-\hat{p_{i}}(c)\right)}^{2} \end{array}$$

where (*x_i_*, *y_i_*, *w_i_*, *h_i_*) is the prediction box, ($\hat{x_{i}}$,$\hat{y_{i}}$,$\hat{w_{i}}$,$\hat{h_{i}}$) is the real box, the first line judges that the box *j* in the grid *i* is responsible for detecting this object and calculating the positioning loss, and the second line is the confidence loss, when there is a target in the grid, it returns to 1, and when it does not exist, it returns to 0. The last line calculates the classification loss.

### 2.3. Multi-Objective Motion Planning of Robot

After obtaining the location information of coal and gangue, if they are sent to the robot controller at the same time, it will cause confusion. However, if sorting in random order, the efficiency is difficult to be guaranteed. According to the CG-YOLO detection results, the targets are sorted by confidence, and the distance between the targets and the end of the robot should be calculated, respectively. The distances between the robot and the targets are different, and the particle size is also different. In addition, the confidence levels returned are not the same. According to the sorting strategy, reasonable motion planning of the robotic arm is designed by separating as much gangue as possible in the shortest possible time. Assuming the robot end coordinates (*x_r_*, *y_r_*, and *z_r_*), the distance between object n with the robot end coordinates is *d_n_*, and the formula is as follows:

(5) $$d_{n}=\sqrt{{\left(x_{r}-x_{o}\right)}^{2}+{\left(y_{r}-y_{o}\right)}^{2}+{\left(z_{r}-z_{o}\right)}^{2}}$$

In order to comprehensively consider the situation of each target, we propose a model of multi-objective motion planning of robot. In this model, the sorting weight guides the robot sorting. For the sorting weight, the distance between the target and the robot, the confidence of the target, and the size of the target will be included. The calculation of sorting weight is as follows:

(6) $$W_{n}=d_{n}+n\times P_{objn}\times w_{n}\times h_{n}$$

**Figure 4:**
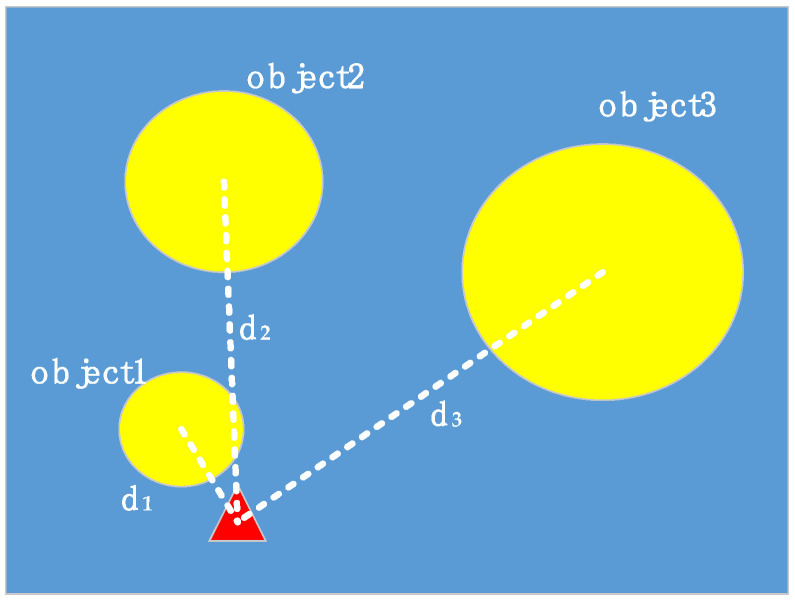
Distribution of objects and robots.

In the formula, *n* is used to adjust the proportion of prediction box. When the value of *n* is larger, the sorting weight is more sensitive to prediction box. For example, there are three targets in the image, but the time is only enough for the robot to perform one movement, as shown in Figure 4.

In Figure 4, the three yellow ellipses are the objects, the red triangle is the initial robot position. The ideal situation is to sort out the largest object (object 3), but if the distance of the largest target is relatively long and the robot trajectory is long, the time for the robot to move to object 3 may not be enough. If only consider sorting the nearest target, it is possible to pick the small one (object 1) and miss the big ones. However, by this model, the optimal solution can be acquired. The value of n should be related to the position and the execution speed of the robot and the speed of the conveyor. In conclusion, sorting weight is an important indicator of robot sorting, which determines the order of sorting, and therefore affects the efficiency of sorting.

## 3. Simulation and Experiment

### 3.1. Simulation

It is very complicated to build and debug the robot system, because the research and development cost is high and the cycle is long. When there are any problems in the basic links, the algorithm cannot be realized. In order to solve this problem, the entire sorting system is modeled in the robot simulator software CoppeliaSim, and the workspace of the robot is calculated, and the layout of the camera, conveyor belt, and robotic arm are adjusted through simulation [32].

**Figure 5:**
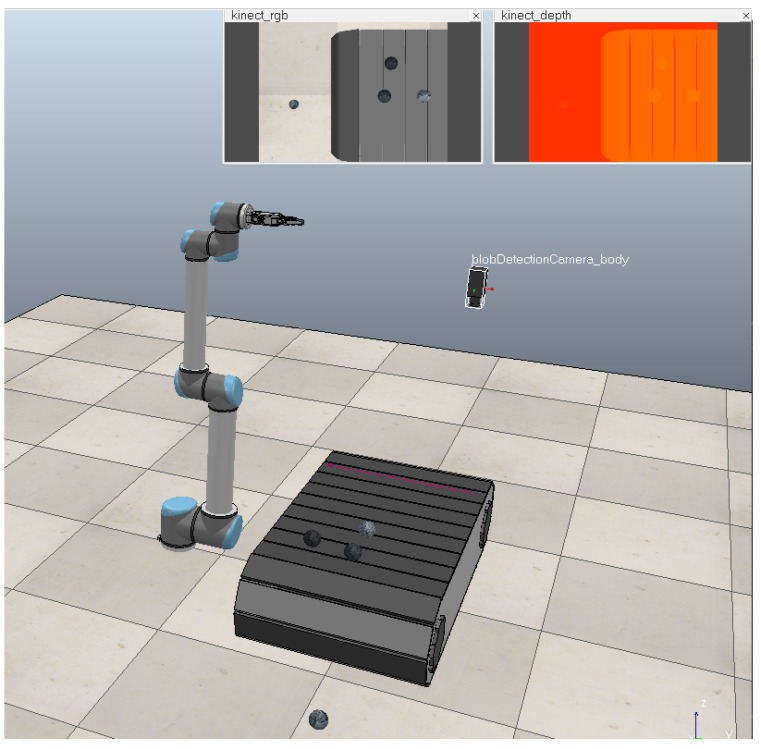
Simulation of coal gangue separating robot system.

The system is composed of target, image collector, conveyor belt, image recognition system, mechanical arm, and robot control system, as shown in Figure 5. In CoppeliaSim, randomly shaped coal and gangue are simplified into spherical shapes, distinguished by different gray levels. Under this simplified model, the location of the target is identified. By setting the range of motion of each joint of the robot, three-dimensional workspace of it can be simulated. The objects are sorted in the workspace, but other parts, such as the camera, need to be placed outside the workspace to ensure safety. In a virtual environment, it is easier and intuitive to realize the adjustment of the target distribution, the speed of the transmission belt, and the movement of the hardware in the system in the space, which has guiding significance for the experiment.

In the simulation, due to the fast response speed of the robot, the communication time is negligible. When the conveyor speed is lower than 70 mm/s and the target number is less than or equal to 3, all objects sorting can be achieved.

### 3.2. Experiment and Result

Some tricky problems in the experiment were solved through simulation, including the position and height matching of conveyor belt, camera, robot body, etc.

**Figure 6:**
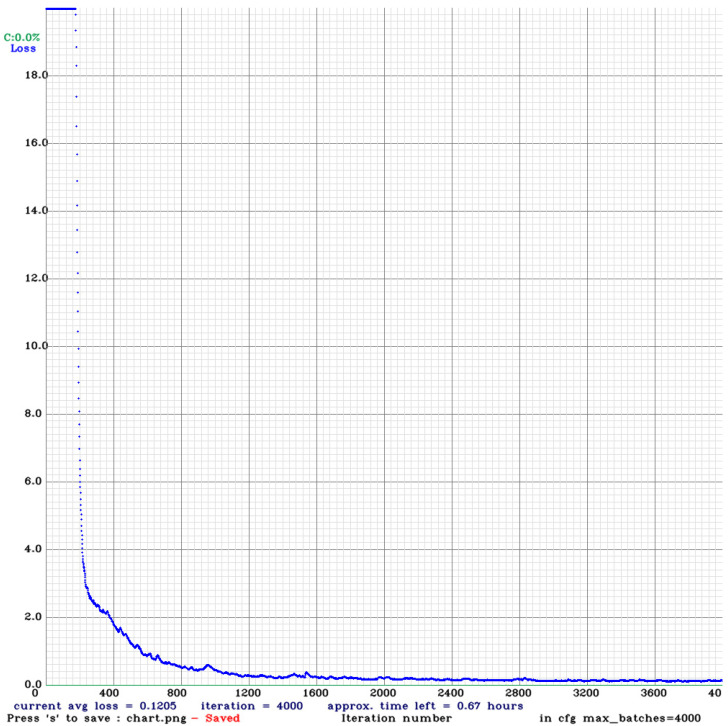
Target detection model training process.

In the target detection experiment, we divide the data set into a training set and a validation set at a ratio of 8:2. The training set contains 1616 images, including 3623 gangue objects and 2312 coal objects. After 4000 rounds of training, the loss is reduced to an acceptable range and the model is obtained, as shown in Figure 6. The trained model is tested by the valid images, and the AP of coal is 98.05%, the AP of gangue is 98.27%, and the mAP of this model is 98.16%. On the basis of already meeting the high recognition accuracy rate, the recognition speed meets the real-time requirements.

The trained model is tested by the valid images (the validation set in the data set), and the AP of coal is 98.05%, and the AP of gangue is 98.27%, and the mAP of this model is 98.16%. On the basis of already meeting the high recognition accuracy rate, the recognition speed meets the real-time requirements.

**Figure 7:**
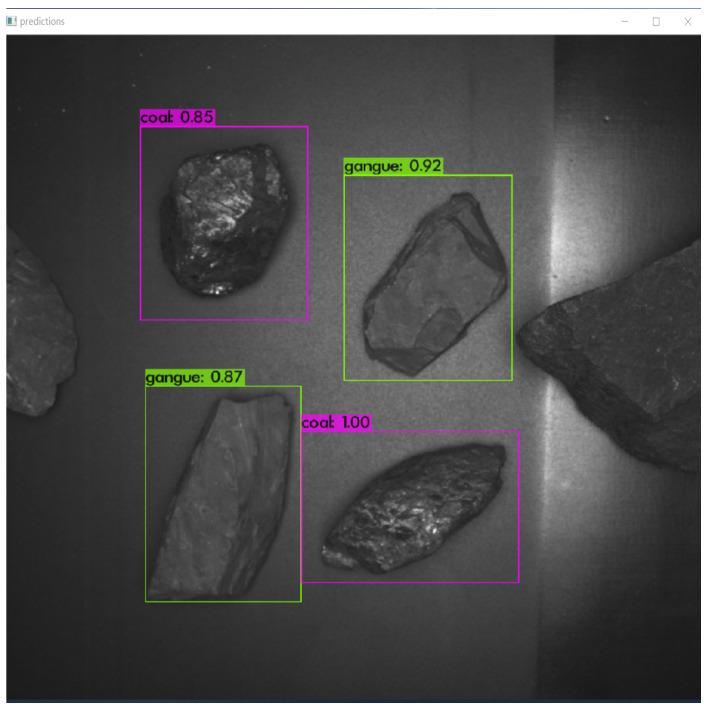
Detection of coal and gangue.

In real-time coal and gangue sorting robot experiment based on target detection, we take the captured picture 2048 × 2048 scaling to the input size of the convolutional network 416 × 416 according to the aspect ratio as input. The frame coordinates obtained through the above network are (*t_x_*, *t_y_*, *b_h_*, and *b_w_*), i.e., the bounding box is relative to the location and size of the feature map and the confidence level. After Non-Maximum Suppression (NMS), predictions with high confidence are left. At the same time, the maximum number of prediction boxes is set to 20. The return information of the prediction box is enlarged to the original picture 2048 × 2048, and the target in the picture is finally detected as shown in Figure 7, and each is returned separately. The confidence levels of the target, the position, and the width and height of the prediction box are as shown in Table 2.

**Figure 8:**
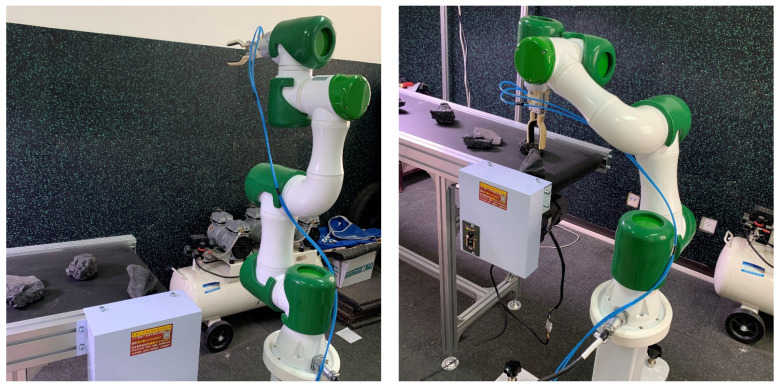
Robot sorting experiment based on multi-objective motion planning model.

Four targets are identified in the image, two coal pieces and two gangues. The scheme of sorting gangue out of the mixture is considered. The gangue identification results are sent to the multi-objective motion planning model for robot motion planning; then, the planned sorting sequence is sent to the robot controller through the serial port to control the robot, as shown in Figure 8. After many experiments, it is found that the number and position of targets in the field of view affect the separating efficiency. When the conveyor belt is at low (10 mm/s), medium (50 mm/s), and high (100 mm/s) speeds, the separating rate of robot using the motion planning algorithm proposed in this paper is shown in Table 3 below.

According to the table, when moving at a low speed and when there are a few sorting targets, whether to plan has little effect on the sorting effect. However, when the conveyor belt moves faster, reasonable planning can ensure that the most cost effective gangue is picked out in a limited time.

## 4. Discussion

In coal production, the separating of coal and gangue is a typical repetitive heavy manual labor. The application of artificial intelligence technology to replace manual labor can liberate workers from harsh working conditions and improve work efficiency. However, in this process, the identification of coal and gangue and the sorting of robots are both difficult. Many studies limit the recognition to the two-class classification of coal and gangue, but classification alone cannot achieve sorting. We proposed the application of computer vision technology to identify coal and gangue. In order to ensure real-time performance, we first train the dataset established by us through YOLO v3. The target detection effect obtained was not ideal. We aimed at the characteristics of coal and gangue and proposed CG-YOLO recognition algorithm; then, CG-YOLO has achieved a recognition accuracy of more than 98%. CG-YOLO has reference significance for target detection such as medical images with similar foreground and background and large differences within the class. Most of the research on coal and gangue sorting focuses on identification problems, but the follow-up still relies on manual separation. As an essential part of coal pre-combustion treatment, it is not enough to identify coal and gangue. A system of hand and eye coordination composed of vision technology and robotics technology can be formed to break the limitations of the application of a single technology, solve problems in industrial production, and promote industry progress. The entire system is a comprehensive application of artificial intelligence technology and has strong portability in various coal mines. Through simple training, even workers who do not understand algorithms can monitor the operation of the system on the upper computer, and therefore workers do not need to work in dangerous and harsh environment, which can reduce casualties. At the same time, the robot applied to the specific scene can work 24 h a day, and the mistakes will be reduced, the work efficiency can be improved, and more benefits could be created for the enterprise.

## 5. Conclusions

In this paper, we presented a complete separating system, not just image recognition like most studies. A target detection algorithm that can recognize and locate coal and gangue is proposed, and this method can achieve high recognition accuracy. According to the detection results, we designed a robot motion planning model, so that the robot can sort out as many gangues as possible in a limited time. In this case, the sorting efficiency is improved. Finally, we do the robot grasping experiment, and get an average grasping rate of 75%, improving the accuracy and efficiency of coal and gangue separating. Although the types of coal and gangue in different regions are not the same and the composition and appearance are slightly different, the method proposed in this article has strong flexibility and versatility. With simple equipment, being environmentally friendly, and nonrequirement of extensive reconstruction of the workshop, this method could be transplanted to other coal mines to solve production problems by the parameter adjustment. Using vision-based robot systems to replace workers in coal production with harsh, dangerous, and repetitive tasks can improve sorting efficiency and ensure economic benefits. In view of the many casualties and safety hazards caused by coal mine production, the development of the coal industry in the future will inevitably develop in the direction of artificial intelligence and robots. The coal gangue robot sorting system based on computer vision proposed in this paper is an application of artificial intelligence and robot technology. The realization of unmanned or less humanized underground operations has important practical significance and is a frontier attempt.

In the next work, we will expand the dataset further, so that the model has better accuracy and robustness. For robots, we will improve the control algorithm from the perspective of dynamics and strive for robot execution speed.

## Figures and Tables

**Table 1:** Distribution of dataset.

	Coal	Gangue	Mix
Low speed	100	200	520
Medium speed	100	200	300
High speed	100	200	300

**Table 2:** Return value of coal and gangue detection.

Objects	Confidence	Horizontal Coordinate	Longitudinal Coordinates	Width	Height
gangue	0.924509	619.060181	265.919861	923.960083	654.644470
gangue	0.865843	260.949890	664.420837	542.986572	1072.973511
coal	0.854325	251.618530	173.845474	554.370850	539.189697
coal	0.998821	542.005005	750.845947	935.131104	1036.864746

**Table 3:** Comparison of sorting rate under motion planning.

Number of Objects	Low Speed	Medium Speed	High Speed
≤2 (with plan)	100%	100%	100%
≤2	100%	100%	100%
3–4 (with plan)	100%	65%	35%
3–4	100%	45%	20%
>4 (with plan)	72%	35%	35%
>4	60%	20%	20%

## Data Availability

Not applicable.
